# Report of Committee on Incorporation

**Published:** 1894-11

**Authors:** 


REPORT OF COMMITTEE ON INCORPORATION, THIRTY-FIRST ANNUAL MEETING, UNITED STATES VETERINARY MEDICAL ASSOCIATION.

PHILADELPHIA, PA., Sept., 1894.

GENTLEMEN:—Your Committee on "Incorporation" beg leave to make the following report of their progress in the attempt to obtain a charter, for the Association, from the Congress of the United States:

This Committee was appointed in September, 1893, but before that time some preliminary work had been done, which consisted of an explanation, to one of the Senators, of what the Association desired to accomplish; and in taking his advice as to the best methods of preliminary procedure. As a result of this and some correspondence, the following letter, dated February 11th, 1893, was received:

"HOUSE OF REPRESENTATIVES, U. S.,

"WASHINGTON, D. C., Feb. 11, 1893.

"MY DEAR SIR:—It is now too late in the session to do anything with any new bill in this Congress, but if you have an act of incorporation drawn, I think there will be no difficulty in getting it through Congress at the next session. These bills are usually passed without objection. Any good lawyer could draw it for you.

"Very truly yours,

"H. C. LODGE."

As well as one dated February 25th, 1893, as follows:

"HOUSE OF REPRESENTATIVES, U. S.,
"WASHINGTON, D. C., Feb. 25, 1893.

"MY DEAR SIR:—If there is an extra session of Congress, I shall be glad to introduce the bill you speak of.

"Very truly yours,
"H. C. LODGE."

After the Committee were appointed, and had some consultation with one another by letter, it was determined to have a meeting, that a fuller interchange of opinion could be had, and, if possible, some plan of operation decided upon. It was further decided to hold this meeting in Philadelphia, so that our President might conveniently be present to aid us with his advice.

At this meeting it was decided to have a proper act of incorporation drawn up by a lawyer, as advised by Senator Lodge; to then send it to him for introduction; and after that to await his further instruction.

This was done, and the following letter was received:

"SENATE CHAMBER,
"WASHINGTON, D. C., Dec. 30, 1893.

"MY DEAR SIR:—I will present and introduce your bill, with pleasure. Whether it will be necessary for your Committee to come here, so far as the Senate is concerned, I do not know. I think probably not. When it gets to the House it will require more looking after to prevent it from being pigeon-holed.

"Truly yours,
"H. C. LODGE."

Shortly after this a copy of the bill, as introduced, was received; it is a Senate bill, No. 1492, and a copy of it accompanies this report.

The matter was then allowed to rest in the hands of Senator Lodge until February, when, nothing further having been heard from it, a letter was written, to which the following reply was received:

"UNITED STATES SENATE,
"WASHINGTON, D. C., Mar. 3, 1894.

"MY DEAR DR. LYMAN:—The bill is in the Committee of Military Affairs. I have spoken to Senator Manderson about it, but have been so busy with other matters that I have not had time to follow it up. You ought to write to the Committeee on

Military affairs. I cannot think that there would be any opposition to the bill. Just now, however, I am so absorbed in the tariff, that it is very difficult for me to give attention to anything, but I will try to do the best I can.

"Very truly yours,

"H. C. LODGE."

A slight further delay being unproductive of visible result, Dr. Dougherty, of the Committee and of Baltimore, went to Washington in the interest of the bill. Of his experiences there he makes the following report:

"I have made three trips to Washington in the interest of the bill. The first I devoted to hunting up my Congressmen, and after a great deal of trouble, found that a Senate Committee had charge. The second, I hunted up the Senators; and the third trip, after some pretty hard work, located the bill in the Committee on "Agriculture and Forestry," where it still remains. I communicated with the Committee, and also with Senator Gorman, without any result. Dr. Hoskins also wrote his Senators. The Tariff Bill was paramount to everything this session. As soon as the Committee acts, I don't think there will be any trouble in passing the bill."

Your Committee, believing that a charter will be of great advantage to the Association, and that it can be obtained with a comparatively small outlay of time and money, most respectfully recommend that the present endeavor be continued; and that the powers given them should also be given to their successors.

CHAS. P. LYMAN, *Chairman.*
WM. DOUGHERTY,
D. J. DIXON.

FIFTY-THIRD CONGRESS, 2D SESSION.

S. 1492.

IN THE SENATE OF THE UNITED STATES,

Jan. 24, 1894.

Mr. Lodge introduced the following bill, which was read twice and referred to the committee on Agriculture and Forestry.

A BILL

TO INCORPORATE THE UNITED STATES VETERINARY MEDICAL ASSOCIATION.

BE IT ENACTED by the Senate and House of Representatives of the United States of America in Congress assembled, That

W. Horace Hoskins, Thomas B. Rayner, of Philadelphia, Pennsylvania; A. W. Clement, of Baltimore, Maryland; T. J. Turner, of Columbia, Missouri; James L. Robertson, A. Liautard and R. S. Huidekoper, of New York City; W. L. Williams, of Bozeman, Montana; A. H. Baker, of Chicago, Illinois; J. F. Winchester of Lawrence, Massachusetts, and F. H. Osgood, of Boston, Massachusetts, and all such persons as shall or may be associated with them and their successors, are hereby created a body politic and corporate in fact and in law by the name of The United States Veterinary Medical Association, and by that name shall have perpetual succession, and shall be able to sue and be sued, plead and be impleaded, defend and be defended, in all courts of law and equity, and may make and have a common seal, and the same alter at their pleasure; and that the object and purposes of the said corporation are to contribute to the diffusion of true science, and particularly the knowledge of veterinary medicine and surgery.

Sec. 2. That the said Association shall have no capital stock. Fees for membership, and annual dues may be assessed as the corporation by its by-laws may determine, which fees and dues shall only be applied to promoting the object and purposes for which the corporation is formed.

Sec. 3. That the officers of the Association shall be a President, Vice-President, Recording Secretary, and Treasurer, all of whom shall be elected by ballot at each anniversary meeting. A Board of Censors, consisting of seven members, shall be appointed by the President of the Association, to serve for one year. The several officers of the Association, with the Board of Censors, shall constitute the Comitia Minora, who shall transact and manage all the affairs of the corporation, and exercise all its powers, subject to the control of the members in general meeting.

Sec. 4. That Congress reserves to itself the right to alter or repeal this act.

